# The ACE and ACTN3 polymorphisms in female soccer athletes

**DOI:** 10.1186/s41021-021-00177-3

**Published:** 2021-02-18

**Authors:** Qi Wei

**Affiliations:** 1 Key Laboratory of General Administration of Sport of China, Hubei Olympic Center , High-tech Road No.1 of the East Lake High-tech Zone, Hubei 430050 Wuhan, China; 2 Hubei Institute of Sports Science, Hubei Olympic Center , High-tech Road No.1 of the East Lake High-tech Zone, Hubei 430050 Wuhan, China

**Keywords:** *ACE*, *ACTN3*, Polymorphism, Female soccer, Sports performance, VO_2_max

## Abstract

**Objects:**

We investigated the association of *ACE* I/D and *ACTN3* R577X polymorphisms with the performance of Chinese elite female soccer athletes for the first time.

**Material and methods:**

The genotype distributions of *ACE* I/D and *ACTN3* R577X in the athlete group and the control group of Chinese females were evaluated via PCR and compared. VO2max value was tested as per standard protocol.

**Results:**

Regarding the distribution of *ACE* polymorphisms, the genotype frequency was indifferent between the athletes (II 40 %, ID 46.7 %, DD 13.3 %) and the controls (II 42 %, ID 48 %, DD 10 %). No difference in the I/D allele frequency was observed between the athlete group and the control group. Regarding the distribution of *ACTN3* polymorphisms, the genotype frequency was significantly different between the athletes (XX 0 %, XR 53.3 %, RR 46.7 %) and the controls (XX 16 %, XR 44 %, RR 40 %). The allele frequency was observed no different between the athlete and the control group. The ACE ID and ACTN3 RR genotype combination was associated with higher VO2max values among defenders than among other players. According to VO2max values,The *ACE* and *ACTN3* genotype combinations (II/ID/DD + RR/XR) significantly differed between the athletes and the controls (*p* < 0.05).

**Conclusion:**

These results suggested that the Chinese elite female soccer athletes were more likely to harbor the I allele and the R allele and that the combination of *ACE* II/ID and *ACTN3* RR/XR was a synergetic determinant of the athletic performance of females in soccer.

## Introduction

With the completion of the Human Genome Map (HGM), the efficiency of human genomics research has been greatly enhanced. Many genetic studies related to sports performance have been performed based on the hypothesis that the human genome contributes to individual physical functions, such as endurance, muscle strength and coordination, equilibrium, flexibility, and even psychological motivation. Sports and physical performance are complicated outcomes determined by the contributions of hundreds of individual genetic polymorphisms and environmental stimuli [30]. Polymorphisms in the angiotensin-converting enzyme (*ACE*) gene and the α-actinin-3 (*ACTN3*) gene have frequently been studied as genetic markers of athletic performance, particularly for power- and endurance-oriented athletic events [2].

The angiotensin-converting enzyme(ACE)is a pivotal constituent of the renin-angiotensin system (RAS), which is responsible for regulating human blood pressure and maintaining circulatory homeostasis via the vasoconstrictor angiotensin II. Thus, the roles of ACE in athletic performance have been widely studied. The *ACE* gene is located on chromosomal region 17q23, and this region contains polymorphisms referred to as the I allele, in which there is an insertion in intron 16 of a 287-bp Alu element, and the D-allele, in which this sequence is absent.

The *ACE* I/D polymorphism is classified into three genotypes, II/ID/DD, and is associated with circulating and tissue ACE levels. An overrepresentation of II subjects was first found in endurance athletes, specifically 25 elite mountaineers [25], followed by elite Australian rowers [14], 34 elite British runners [26], Spanish elite cyclists, long-distance runners, and handball players [5], Russian [27] and South African triathletes [10], and Russian rowers [3]. The association between the *ACE* genotype and endurance performance among Asians was demonstrated in a meta-analysis [20]. Conversely, the D allele has been strongly demonstrated to provide greater strength, including increased muscle volume and an increased percentage of fast-twitch muscle fibers. Many studies showed the D allele to be overrepresented among power athletes, as observed in 20 British [26], 65 Russian [27], and 56 European Caucasian swimmers [39]. Gineviciene et al. found that the DD genotype and the D allele were clearly less frequent among outstanding power-oriented athletes than among the national population and a control group [15]. Subsequently, Wang et al. reported that 166 East Asian short-distance swimmers more commonly carried the ACE I allele than controls [38].

The *ACTN3* gene encodes the Actn3 protein in skeletal muscle, which is a structural constituent of the Z disc. Actn3 is crucial for establishing the structure and regulating the cytoskeletal organization of type II fibers. The polymorphism of the *ACTN3* gene converts a C to a T at nucleotide position 1747 of exon 16, leading to a mutation of codon 577, replacing an arginine-coding codon (R allele) with a termination codon (X allele). Previous studies presented strong evidence that the *ACTN3* RR genotype is associated with sprint-/power-oriented sports performance [21, 28, 41] and that the R allele correlates with high-power muscle contractions and an increased relative surface area and number of fast-twitch muscle fibers [37]. The presence of the R allele was recently found to be associated with performance on sprint/power athletic events based on a meta-analysis. XX homozygotes expressed no Actn3 protein in muscle, and this genotype was associated with elite endurance performance among athletes from different populations, such as elite Australian athletes [41], Finnish athletes [28], and Israeli top-level athletes [40]. These findings suggested that the absence of Actn3 protein could be conducive to endurance activity [41]. In contrast, other authors found high-level sprint-/power-oriented Olympic athletes carrying the XX genotype [11]. Based on an Actn3 knockout mouse model, the *ACTN3* genotype shifted towards the X allele during evolution, leading to diminish the muscle mass and fiber diameter, reduced contractility, enhanced resistance to fatigue, and elevated oxidative enzyme activity. These results reflect that the absence of the Actn3 protein could have a negative effect on fast-twitch skeletal muscle fiber functions [40]. These data provide some insight into the mechanisms underlying the associations of power athlete status with Actn3 deficiency and skeletal muscle hypertrophy [1].

The review has shown that genetic association research in soccer predominately focused on the *ACTN3* R577X and *ACE* I/D polymorphisms increasing at a substantial rate in the last 10 years[22].These studies concertrated on males and Caucasian populations, but less on females and other regions.Our study was aimed to the sample with same gender and ethnicity and investigated the potential correlations of the *ACE* I/D and *ACTN3* polymorphism and sport performance of Chinese elite female soccer athletes firstly .

## Methods

 This approved study was approved by the Sports Medicine Committee at Hubei Sports Science Society. Written informed consent was obtained from each participant and Exercise testing informed consent was signed by each athlete. The study complied with the guidelines set out in scientific research policy of the Key Laboratory of General Administration of Sport of China and referenced the STREGA guidelines to provide below information of the items on the STROBE checklist [19].

### Paticipants

We obtained samples from 60 Chinese female athletes (age 23.8 ± 1.83 years, height 168.3 ± 7.64 cm, and weight 60.75 ± 3.69 kg) and from a control group of 200 common Chinese females (age 18–25 years, height 160.1 ± 5.24 cm, and weight 55.85 ± 2.79 kg).

The female athletes came from Wuhan Jianghan women’s professional football club and Jiangsu Suning ladies football club. The control group came from faculty of management of Huazhong University of Science and Technology(HUST) .The athletes’ group and control group were the Han nationality from southern China.

### Genotyping

Each DNA sample was extracted from peripheral blood using a blood genomic DNA Extraction Kit (Aidlab Co., China).The *ACE* and *ACTN3* polymorphisms were amplified via polymerase chain reaction (PCR) in a total reaction volume of 25 µL containing template DNA (50 ~ 80 g/L), 10x KOD Buffer, KOD-plus polymerase, primers, 2 mMdNTPs, 1.5µL of 25 mM MgCl2, 0.5 U of Taq DNA polymerase, and 16 µL of deionized water. Genotyping was performed according to the amplification protocols in Table 1.

**Table 1:** The amplification protocols for detection of the *ACE* I/D and *ACTN3* polymorphisms

	*ACE* I/D			*ACTN3*		
Protocol	denaturation	95℃	3 min	denaturation	95℃	3 min
	denaturation	95℃	30s	denaturation	95℃	30s
	annealing	60℃	30s	annealing	68℃	30s
	extension	72℃	8 min	extension	68℃	8 min

**Fig. 1:**
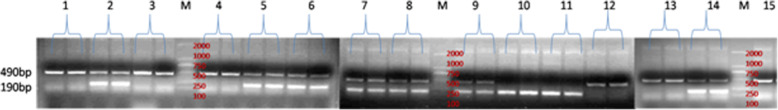
Validation of the I/D polymorphisms of *ACE* via electrophoresis. Notes: M: DNA marker (2000, 1000, 750, 500, 250, and 100 bp); II: lanes 1, 3, 4, 12, 13, and 15; ID: lanes 2, 5, 6, 7, 8, 9, and 14; DD: lanes 10 and 11

PCR was used to detect the I and D alleles of the *ACE* gene according to a previously described method using the upstream primer 5´-CTGGAGACCACTCCCATCCTTTCT-3´ and the downstream primer 5´-GATGTGGCCATCACATTCGTCAGAT-3´ [34]. The reaction products were detected via 6 % polyacrylamide gel electrophoresis. The PCR fragments were detected as follows: DD, 190 bp fragment; II, 490 bp fragment; ID, 490 and 190 bp fragments (Fig. 1).

**Fig. 2:**
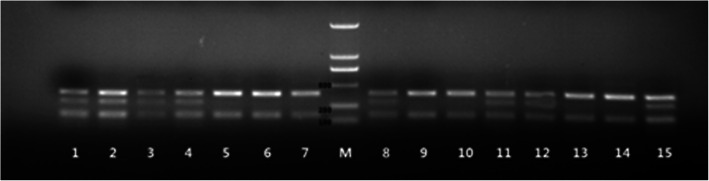
Validation of the R577X polymorphism of ACTN3 via electrophoresis. Notes: M: DNA Marker (2000, 1000, 750, 500, 250, and 100 bp); XR: lanes 1, 2, 3, 4, 8, 11, 12, and 15; RR: lanes 5, 6, 7, 9, 10, 13, and 14

**Fig. 3:**
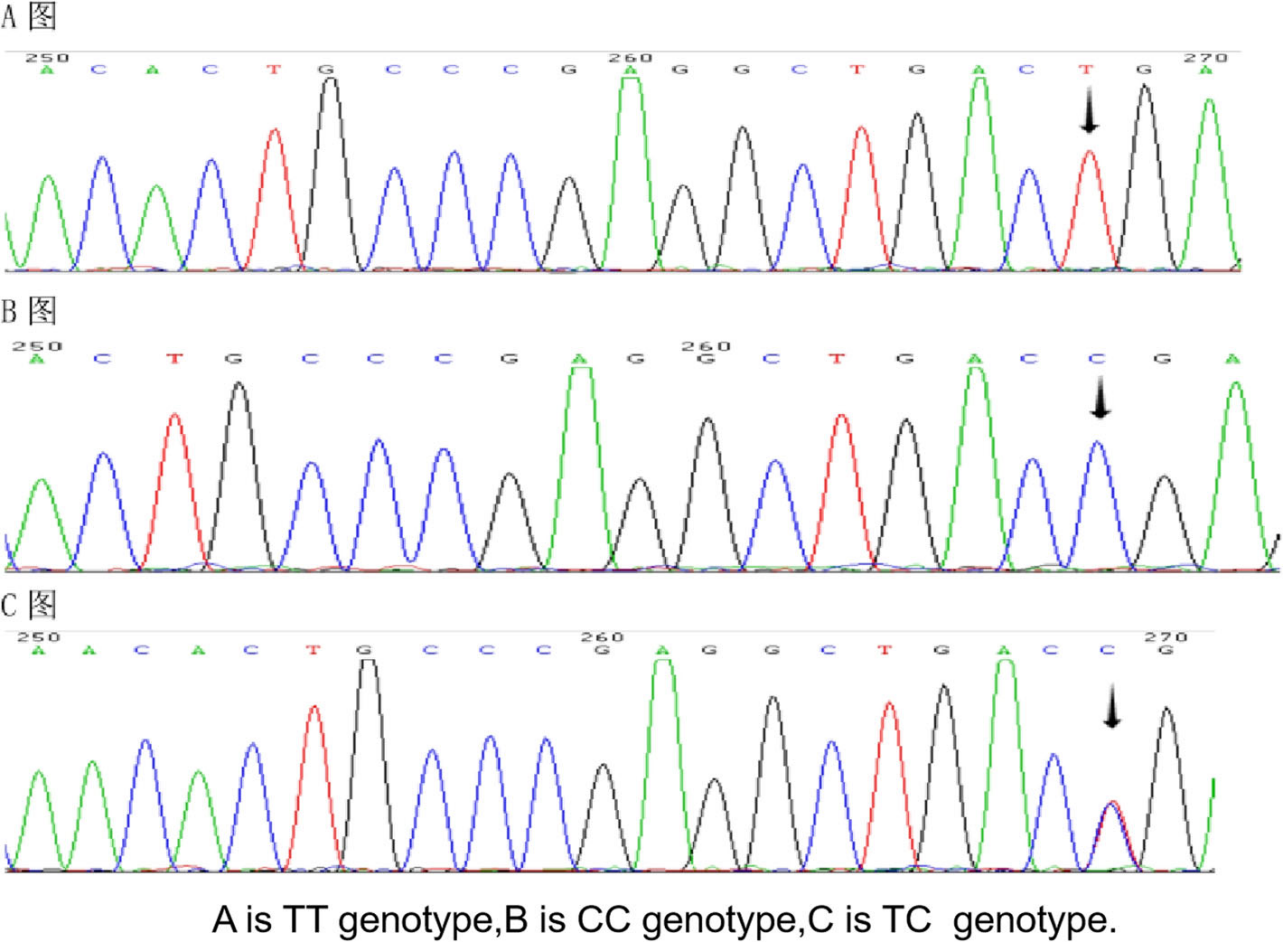
Sequence diagram of ACTN3 R577X PCR digestion product by ABI 3730 DNA Analyzer. Notes: **a**: TT genotype, **b**: CC genotype, **c**: TC genotype

The ACTN3 R/X polymorphism was amplified via PCR using the forward primer 5-CTGTTGCCTGTGGTAAGTGGG-3´ and the reverse primer 5-TGGTCACAGTATGCAGGAGGG-3´. The amplified fragment was subsequently digested with the restriction enzyme Dde I as described by Mills et al. [24]. Allele X shows two fragments at 205 and 86 bp, whereas allele R shows three fragments of 108, 97, and 86 bp (Fig. 2).The digested PCR fragments were sequenced for identification of ACTN3 using an ABI 3730 DNA Analyzer (Fig. 3).

### Statistical analysis

The genotype and allele frequencies were evaluated for compatibility with Hardy-Weinberg equilibrium (HWE). Statistical analyses of genotype frequencies and allele frequencies were performed using χ2 tests to compare the athlete and control groups. The level of significance was set to p < 0.05. IBM SPSS Statistics 21 software (SPSS Inc., Chicago, IL, USA) was applied for statistical analysis.

## Results

The distributions of the *ACE* and *ACTN3* polymorphisms were compared between 60 Chinese female soccer athletes and 200 healthy female non-athletes. The data for the genotype and allele distributions are listed in Table 2.

**Table 2:** Distributions of the *ACE* I/D and *ACTN3* gene polymorphisms in elite female soccer players and non-athletes from China

Groups		*ACE* Genotype Frequency(%)			Allele Frequency		*ACTN3* Genotype Frequency(%)			Allele Frequency	
	N	II	ID	DD	I	D	XX	XR	RR	X	R
Controls(F)	200	84(42 %)	96(48 %)	20(10 %)	132(66 %)	68(34 %)	32(16 %)	88(44 %)	80(40 %)	76(38 %)	124(62 %)
Athletes(F)	60	24(40 %)	28(46.7 %)	8(13.3 %)	38(63.3 %)	22(36.7 %)	0^a^	32(53.3 %)^a^	28(46.7 %)^a^	16(26.7 %)	44(73.3 %)
Total	260	108(41.5 %)	124(47.7 %)	28(10.8 %)	170(65.4 %)	90(34.6 %)	32(12.3 %)	120(46.2 %)	108(41.5 %)	92(35.4 %)	168(64.6 %)

Notes:^a^χ2 = 9.864, df = 2, *p* = 0.007 for the comparison of the ACTN3 genotype frequencies between the the athletes and the control subjects

The *ACE* genotype showed no deviation from Hardy-Weinberg equilibrium in the athlete group (II/ID/DD,40 %/46.7 %/13.3 %;χ2 = 0.00137, *p* = 0.97)or the control group (II/ID/DD, 42 %/48 %/10 %;χ2 = 0.96, *p* = 0.32). The genotype and allele frequencies of *ACE* I/D no differed significantly between the athlete and control groups (χ2 = 0.187, df = 2, *p* = 0.911;χ2 = 0.055, df = 1, *p* = 0.815, respectively).

The distribution of the *ACTN3* R577X genotype among the female athletes was 53.3 % carrying the XR genotype, 46.7 % carrying the RR genotype, and none carrying the XX genotype (χ2 = 0.793, *p* = 0.159). Alternatively, this distribution among the controls was 12.3 % carrying the XX genotype and 46.2 % carrying the RR and 41.5 % carrying the XR genotypes (χ2 = 0.02, *p* = 0.88). The genotype frequencies of *ACTN3* R577X differed significantly for the comparison between the female athletes and the controls (χ2 = 9.864, df = 2, *p* = 0.007). The allele frequencies of *ACTN3* R577X were not different between the athlete and control groups (χ2 = 0.101, df = 1, *p* = 0.751).

According to their position in the field, the athletes in our cohort were categorized as forwards, defenders, midfielders and goalkeepers. The results of genotype combinations and VO_2_max values for each position are listed in Table 3. This analysis revealed that the *ACE* II and *ACTN3* XR combination and the *ACE* ID and *ACTN3* RR combination were overrepresented relative to the other combinations. The *ACE* ID and *ACTN3* RR genotype combination was associated with higher VO_2_max values among defenders than among other players. According to VO_2_max values, The *ACE* and *ACTN3* genotype combinations (II/ID/DD + RR/XR) showed significant differences between the athletes and the controls based on the χ2 test (*p* < 0.05), as shown in Table 3.

**Table 3:** The *ACE* and *ACTN3* genotype combinations and VO_2_max values for players of different positions

Positions	*ACE* and *ACTN3* Genotype Combinations							VO_2_max (ml/kg/min)
	II + RR	II + XR	ID + RR	ID + XR	DD + RR	DD + XR	others	
Forwards	0	12	4	4	4	0	0	51.05 ± 2.61
Midfielders	0	4	12	4	0	4	0	51.74 ± 3.88
Defenders	0	0	4	0	0	0	0	54.14 ± 2.39
Goalkeepers	4	4	0	0	0	0	0	52.11 ± 2.14
All athletes	4^a^	20^a^	20^a^	8^a^	4^a^	4^a^	0	52.66 ± 3.36
Controls	44	32	12	8	4	4	96	

Notes: ^a^χ2 = 11.952, df = 5, *p* = 0.035 for the comparison of the *ACE* and *ACTN*3 genotype combinations between all athletes and the control subjects

## Discussion

Since 1998, the *ACE* I/D polymorphism has become a gene marker widely associated with human physical performance [25]. In subsequent years, at least 79 genes have been found to be on loci related to elite athlete status [2]. The I allele was associated with optimal vascular tension, dominance of slow-twitch muscle fibers, and amenability to enhanced aerobic endurance; alternatively, the D allele was associated with increased *ACE* activity and abundance of fast muscle fibers beneficial to the development of speed–strength qualities, as well as predisposition for arterial hypertension and myocardial hypertrophy [14, 25–27]. Various *ACE* gene polymorphisms can affect muscle mass and physical strength [17]. Taylor concluded that the distribution of *ACE* genotypes could be distinct between males and females [35]. The previously investigated associations of *ACE* and *ACTN3* polymorphisms with athletic performance were debatable due to the races, geographical locations, genders, athletic statuses and sports disciplines studied. One author analyzed the data from 25 published studies of *ACE* and 28 published studies of *ACTN3* via a meta-analysis stratified according to gender, ethnicity and sports discipline (p < 0.05). The results established that the II genotype of *ACE* is related to endurance performance and that the *ACTN3* R allele is related to power performance [20].Based on the meta-analysis of football, Significant associations were shown between the presence of the ACTN3 R allele and professional footballer status and the ACE D allele and youth male footballers [22].

Therefore, we aimed to reveal the interactions of genetic factors, environmental factors and training stimuli on female physical phenotypes while eliminating the interference of gender. We chose a sample of elite soccer players with many years of training. This study individually explored the associations of *ACE*I/D and *ACTN3* polymorphisms with athletic performance. The results showed that the genotype frequencies of *ACE* ID and DD were the highest and lowest, respectively, among female soccer players and that the frequencies of both genotypes were significantly different between the athletes and the controls. Micheli et al. reported that the ID genotype played a critical role in determining enhanced athletic performance on the squat jump and the countermovement jump and optimal body composition among young soccer players [23]. Spanish soccer players exhibited an increased frequency of the *ACE* ID genotype compared to the general population [15]. Juffer et al. demonstrated that the II genotype was rare among soccer players in Lithuania and that the ID genotype was more frequent among these soccer players than among controls [18]. However, Daniel et al. analyzed the allele and genotype frequencies of *ACE* I/D and showed no significant difference between Brazilian soccer players and controls [9].

The results of our investigation of *ACE* I/D polymorphisms among female soccer players revealed a high frequency of the I allele, and this finding validates the results of these previous studies. Therefore, our results indicate that the studied Chinese female athletes were predisposed to aerobic endurance in soccer.

The Actn3 protein constitutes the Z line of the skeletal muscle structure. In 2003, Yang et al. clearly demonstrated the *ACTN3* R allele was relevant to elite power/sprint performance and that the X allele was relevant to endurance performance; in particular no XX genotype carriers found among the studied females [41].

It has been reported that the *ACTN3* XX genotype was notably less frequent in a groups of Finnish sprinters [27], although identical distributions were observed in Russian power-oriented athletes [11] and Italian artistic gymnasts compared to controls [21]. Although some authors reported that the X allele is related to athletic endurance based on case-control studies [13, 33, 41], the majority of results have shown no such association [28, 36]. It is difficult to unequivocally prove that carrying the X allele is conducive to athletic endurance.

The *ACTN3* RR genotype was associated with power/sprint event performance among Israeli sprinters [13] and Russian short-distance skaters [2]. Alfred et al. reported that European power athletes harbored the RR genotype more frequently than the general population based on a meta-analysis[4]. Young healthy men carrying the RR genotype possessed a higher cross-sectional area and a greater number of type II (fast-twitch glycolytic) fibers than those carrying the XX genotype [37]. In a replicated study, a cohort of men and speed skaters carrying the XX genotype exhibited a higher percentage of slow-twitch muscle fibers [2]. Additionally, it should be mentioned that other studies have produced negative results regarding the link between the *ACTN3* R allele and power athletic performance [15, 41].

Among our athletes, the *ACTN3* genotype was either XR or RR, and no XX genotype carriers were observed, as presented in Table 2. These results suggested that the Chinese elite female soccer players harbored a power-/strength-oriented genotype. Previous articles reported a high distribution of the R allele among male soccer players from Spain [32] as well as professional Brazilian (RR, 45 %; RX, 44 %; XX, 11 %) [30], Russian (RR, 46.25 %; RX, 42.5 %; XX, 11.25 %) [12], and young Brazilian soccer players [29].

Investigators have previously explored the mechanism underlying the interactions between various genes and athletic performance, and much evidence has been produced. Genes associated with fast-twitch muscle fibers were overrepresented among Lithuanian soccer players [15]. Compared to those harboring the XX genotype, Brazilian soccer players harboring the *ACTN3* RR/RX genotype exhibited superior strength [30], higher levels of testosterone and interleukins and less vulnerability to accidental training injury [29]. All of these studies were focused on male soccer players, and the results supported the notion of a tendency toward a power-/strength-oriented genotype.

It has widely been established that the aerobic energy pathway predominates among soccer players during a match, consisting of sprints, jumps, heading, dribbling, and shots on goal, with varying speed. Due to these characteristics of a soccer match, maximizing oxygen uptake and strength are important for soccer players to perform at high levels [18].Recently, the *ACTN3* R allele was observed to be related to high levels of testosterone in both male and female athletes [1]. An analysis reported that athletes harboring the XX genotype might not be capable of the physical demands of modern soccer, including high speed and strength. For instance, these players might require more time to recover from match or training fatigue [8]. A very recent study noted that the RR genotype was associated with elevated expression of the Actn3 protein, increased muscle volume, accelerated contraction velocity of muscle fibers, and enhanced explosive contractions [6]. These observations may explain the partial connection between the RR genotype, skeletal muscle hypertrophy, and the status of power/sprint athletes. Another study showed different results: Shang et al. found that the XX genotype was highly represented among female, but not male, elite endurance athletes in China [33].

Summarizing the above findings and our experimental evidence, the R allele was dominant in the athlete group. This result implied that the studied Chinese female athletes were conferred with power/sprinting abilities together with skeletal muscle hypertrophy that beneficial to soccer performance.

With the gradual progression of human genomic functional research, the focus has shifted from genetic factors to gene polymorphisms and their underlying mechanisms related to potential athletic endurance and strength trainability. Accumulating researchers are paying great attention to the cumulative effect of genotypes on human physical phenotypes, aiming to identify the optimal genotypes for sports performance. Analysis of genotype combinations is a very useful method for identifying influences on metabolism. Several articles have reported positive associations between specific gene polymorphisms and the status of a soccer player. For instance, Santiago illustrated that compared to runners, elite soccer players more frequently exhibited the power/sprint performance-related genotypes of RR or RX as well as the ID genotype rather than the II genotype of *ACE* [32]. Micheli et al. deduced that the *ACE* and VDR genotypes might predispose young athletes with high-potential genetic backgrounds to achievement in soccer [23]. Regarding the performance of Lithuanian soccer players, either *ACE* ID or *ACE* II combined with *PPARA* GG, *PPARGC1A* GG was a preferable genotype [15]. The *ACE* DD genotype (60 %) and the *ACTN3* RR genotype (63 %) were most frequently expressed among male Italian soccer players, although no significant differences in the frequencies of these genotypes were observed between these athletes and controls [21]. Extending these results to gene combinations, a recent article showed that Russian soccer players of different positions harbored unique genetic backgrounds; for instance, goalkeepers were more likely to carry the *ACE* D allele, and attacking players were more likely to carry the *ACTN3* R allele [12].

Considering these studies, our study focused on an analysis of the association of *ACE/ACTN3* genotype combinations on human phenotypes. In our study, five of the players harbored the II + XR genotype combination, and the same number harbored the ID + RR genotype combination. On the other hand, one player each harbored the II + RR genotype combination and the DD + RR/XR genotype combination (Table 3). As soccer players apply a mixture of anaerobic and aerobic energy processes, the VO_2_max values indicated that the genotype combination that predisposed the soccer players to enhanced performance was ID + RR, and this finding suggested that the predominance of the D allele and the R allele conferred a high VO_2_max value, reflecting good endurance and explosive force. VO_2_max is a recognized index of aerobic capacity with high heritability. However, VO_2_max was not convincingly related to the *ACE* genotype of athletes, although this parameter was significantly different between postmenopausal and sedentary samples from European females [31]. Another study found that Chinese young men carrying the D allele exhibited higher VO_2_max values than those carrying the I allele [42].

Further, an analysis of gene interactions showed that the carriers of at least one I allele in the soccer group exhibited higher physical performance if they also possessed at least one R allele (II + XR, ID + RR). Surprisingly, a combined homozygous genotype of *ACE* I/D and *ACTN3* R577X (II + RR) was not observed in the studied soccer players. This result suggests that the *ACE* II genotype might improve endurance performance potential but suppress training-mediated enhancements of muscle mass and strength but that the RR genotype might stimulate fast-twitch skeletal muscle fiber activity to help reach the required speed and muscle contraction intensity. The R allele appeared to counteract the effect of the I allele on the elite female soccer players, who more frequently harbored the power-/speed-oriented heterozygous ID genotype than the controls.

We examined the gene-gene interactions according to soccer position (i.e., forward, midfielder, defender, and goalkeeper), enabling us to ascertain the effects of genotype combinations that might have been hidden otherwise. Our results demonstrated that soccer athletes of different positions harbored different genotypes (Table 3). Along these lines, a recent study showed that internal position differences showed variations in genetic demands similar to differences in combined genetic demands between sports [16]. Clos E et al. also found Goalkeepers, central defenders and central midfielders had a significantly different allele distribution compared with wide midfielders and forward players because of different positon players needing different physical efforts and conditioning [7].

Our study considered the gender, regional location, and race of the cohort, although the samples tested still have limitations such as the limited sample size,the diversity of metabolic pathways and lack of multiple tests. It has been widely recognized that an elite athletes with excellent genetic traits should be continuously prepared via external or internal training to encourage one other to improve on their elite athletic performance. Our current progress only indicates the genetic factors that affect the athletic performance of female soccer players from China. We evaluated the combined impact of *ACE* and *ACTN3* polymorphisms on endurance capacity, but the results remained inconsistent with the findings reported in other controversial articles. In future studies, we should expand the sample size of athletes of the same sport and gender and should measure multiple candidate genotypes/phenotypes to evaluate their associations with sporting or physical activities.

## Conclusions

In this study, we explored the distribution of *ACE*I/D and *ACTN3* R577X polymorphisms among Chinese elite female soccer athletes for the first time. These polymorphisms have been examined via PCR, and we compared the genotype and allele frequencies using a case-control study design. The *ACE* ID genotype and the *ACTN3* XR genotype dominated in the athletes compared to the controls. The I allele was overrepresented without significance, but the R was significantly overrepresented among the athletes compared to the controls. According to previous and our significant results (*p* < 0.05)of the combined effect of these polymorphisms, we hypothesize that elite female soccer players are more likely to harbor the heterozygous genotypes of II + XR or ID + RR, which may be beneficial to endurance and power/speed. Considering the complexity of success in sports, further research into sports genomics is needed to comprehend the genetic composition and molecular physiology of top-level athletes, to help guide the development of sports training, and to promote the advancement of human physical performance.

## Data Availability

The data used and/or analyzed during the current study are available from the corresponding author on reasonable request.

## Abbreviations

ACE: Angiotensin-converting enzyme
RAS: Renin-angiotensin system
ACTN3: α-actinin-3
VO_2_max: Maximal oxygen consumption
PCR: Polymerase chain reaction
HUST: Huazhong University of Science and Technology
HWE: Hardy-Weinberg equilibrium
VDR: Vitamin D Receptor
PPARA: Peroxisome Proliferator Activated Receptor α
PPARGC1A: Peroxisome Proliferator Activated Receptor gamma-coactivator 1 α

## References

[1] Ahmetov II, Donnikov AE, Trofimov DY (2014). Actn3 genotype is associated with testosterone levels of athletes. Biol Sport.

[2] Ahmetov II, Fedotovskaya ON (2015). Current progress in sports genomics. Adv Clin Chem.

[3] Ahmetov II, Popov DV, Astratenkova IV, Druzhevskaya AM, Missina SS, Vinogradova OL, Rogozkin VA (2008). The use of molecular genetic methods for prognosis of aerobic and anaerobic performance in athletes. Hum Physiol.

[4] Alfred T, Ben-Shlomo Y, Cooper R. Hardy Rebecca, Cooper Cyrus, Deary IJ, Gunnell David Harris SE, Kumari M, Martin RM, Moran CN, Pitsiladis YP, Ring SM, Sayer AA, George Smith GD, Starr JM. Kuh Diana, Day I N M, the HALCyon study team.ACTN3 genotype, athletic status, and life course physical capability: meta-analysis of the published literature and findings from nine studies. Hum Mutat. 2011;32(9):1008–18. doi:10.1002/humu.21526.. 10.1002/humu.21526 PMC3174315 21542061

[5] Alvarez R, Terrados N, Ortolano R, Iglesias-Cubero G, Reguero JR, Batalla A, Cortina A, Fernández-García B, Rodríguez C, Braga S, Alvarez V, Coto E (2000). Genetic variation in the renin-angiotensin system and athletic performance. Eur J Appl Physiol.

[6] Broos S, Malisoux L, Theisen D, Thienen RV, Ramaekers M, Jamart C, Deldicque L, Thomis MA (2016). Francaux M. Evidence for ACTN3 as a Speed gene in isolated human muscle fibers. PLOS ONE.

[7] Clos E, Pruna R, Lundblad M, Artells R, Maffulli N. ACTN3’s R577x Single Nucleotide Polymorphism Allele Distribution Differs Significantly in Professional Football Players According to their Field Position. Med Princip Pract.2020.doi: 10.1159/000509089. 10.1159/000509089 PMC7923889 32492691

[8] Coelho DB, Pimenta E, Rosse IC, Veneroso C, Baker KL, Carvalho MR, Pussieldi G, Siami-Garcia E (2016). The alpha-actinin-3 R577X polymorphism and physical performance in soccer players. J Sports Med Phys Fitness..

[9] Coelho DB, Pimenta E, Rosse IC, Veneroso C, Pussieldi G, Baker KL, Carvalho MR, Siami-Garcia E (2016). Angiotensin-converting enzyme (ACE-I/D) polymorphism frequency in Brazilian soccer players. Appl Physiol Nutr Metab.

[10] Collins M, Xenophontos SL, Cariolou MA (2004). The ACE gene and endurance performance during the South African ironman triathlons.MedSci. Sports Exerc.

[11] Druzhevskaya AM, Ahmetov II, Astratenkova IV, Rogozkin VA (2008). Association of the ACTN3 R577X polymorphism with power athlete status in Russians. Eur J Appl Physiol.

[12] Egorova ES, Borisova AV, Mustafina LJ, Arkhipovaa AA, Gabbasova RT, Druzhevskayab AM, Astratenkovab IV (2014). Ahmetova II. The polygenic profile of Russian football players. J Sports Sci.

[13] Eynon N, Banting LK, Ruiz JR (2014). ACTN3 R577X polymorphism and team-sport performance: a study involving three European cohorts. J Sci Med Sport.

[14] Gayagay G, Yu B, Hambly B, Boston T, Hahn A, Celermajer DS, Trent RJ (1998). Elite endurance athletes and the ACE I allele–the role of genes in athletic performance. Hum Genet.

[15] Gineviciene V, Jakaitiene A, Tubelis L, Kucinskas V (2014). Variation in the ACE, PPARGC1A and PPARA genes in Lithuanian football players. Eur J Sport Sci.

[16] Heffernan SM, Kilduff LP, Erskine RM, Day SH, McPhee JS, McMahon GE, Stebbings GK, Neale JP, Lockey SJ, Ribbans WJ, Cook CJ, Vance B, Raleigh SM, Roberts C, Bennett MA, Wang G, Collins M, Pitsiladis YP, Williams AG (2016). Association of ACTN3 R577X but not ACE I/D gene variants with elite rugby union player status and playing position. Physiol Genomics.

[17] Jones A, Woods DR (2003). Skeletal muscle RAS and exercise performance. Int J Biochem Cell Biol..

[18] Juffer P, Furrer R, González-Freire M (2009). Genotype distributions in top-level soccer players: a role for ACE?. Int J Sports Med.

[19] Little J, Higgins JPT, Ioannidis JPA, Moher D, Gagnon F, Moh D, Gagnon F, Elm Ev, Khoury MJ, Cohen B, Davey-Smith G, Grimshaw J, Scheet P, Gwinn M, Williamson RE, Zou GY, Hutchings K, Johnson CY, Tait V, Wiens M, Golding J, Duijn C, v,McLaughlin J,Paterson A, Wells G, Fortier I, Freedman M, Zecevic M, King R, Infante-Rivard C (2009). Stewart A,Birkett N. Strengthening the REporting of Genetic Association Studies (STREGA)—An extension of the STROBE Statement. PLoS Med.

[20] Ma F, Yang Y, Li X, Zhou F, Gao C, Li MF, Gao L (2013). The association of sport performance with ACE and ACTN3 genetic polymorphisms: a systematic review and meta-analysis. PLOS ONE.

[21] Massidda M, Corrias L, Scorcu M, Vona G, Calò MC (2012). ACTN3 and ACE genotypes in elite male Italian athletes. Anthropol Rev.

[22] Mcauley ABT, Hughes DC,Tsaprouni LG,Varley I,Suraci B, Roos. TR,Herbert AJ,Kelly AL.The association of the ACTN3 R577X and ACE I/D polymorphisms with athlete status in football: a systematic review and meta-analysis.2020.doi: 10.1080/02640414.2020.1812195. 10.1080/02640414.2020.1812195 32856541

[23] Micheli ML, Gulisano M, Morucci G, Punzi T, Ruggiero M, Ceroti M, Marella M, Castellini E, Pacini S (2011). Angiotensin-converting enzyme/vitamin D receptor gene polymorphisms and bioelectrical impedance analysis in predicting athletic performances of Italian young soccer players. J Strength Cond Res.

[24] Mills M, Yang N, Weinberger R, Vander Woude DL, Beggs AH, Easteal S, North K (2001). Differential expression of the actin-binding proteins, alpha-actinin-2 and – 3, in different species: implications for the evolution of functional redundancy. Hum Mol Genet.

[25] Montgomery HE, Marshall R, Hemingway H, Myerson S, Clarkson P, Dollery C, Hayward M, Holliman DE, Jubb M, World M, Thomas EL, Brynes AE, Saeed N, Barnard M, Bell JD, Prasad K, Rayson M, Talmud PJ, Humphries SE (1998). Human gene for physical performance. Nature.

[26] Myerson S, Hemingway H, Budget R, Martin J, Humphries S, Montgomery H (1999). Human angiotensin I-converting enzyme gene and endurance performance. J Appl Physiol.

[27] Nazarov IB, Woods DR, Montgomery HE, Shneider OV, Kazakov VI, Tomilin NV, Rogozkin VA (2001). The angiotensin converting enzyme I/D polymorphism in Russian athletes. Eur J Hum Genet.

[28] Niemi AK, Majamaa K (2005). Mitochondrial DNA and ACTN3 genotypes in Finnish elite endurance and sprint athletes. Eur J Hum Genet.

[29] Pimenta EM, Coelho DB, Cruz IR, Morandi RF, Veneroso CE, de Azambuja Pussieldi G, Carvalho MR, Silami-Garcia E, De Paz Fernández JA (2012). The ACTN3 genotype in soccer players in response to acute eccentric training. Eur J Appl Physiol.

[30] Pimenta EM, Coelho DB, Veneroso CE, Barros Coelho EJ, Cruz IR, Morandi RF, De A, Pussieldi G, Carvalho MR, Garcia ES, De Paz Fernández JA (2013). Effect of ACTN3 gene on strength and endurance in soccer players. J Strength Cond Res.

[31] Puthucheary Z, Skipworth JR, Rawal J, Loosemore M, Van Someren K, Montgomery HE (2011). Genetic influences in sport and physical performance. Sports Med.

[32] Santiago C, González-Freire M, Serratosa L, Morate FJ, Meyer T, Gómez-Gallego F, Lucia A (2008). ACTN3 genotype in professional soccer players. Br J Sports Med.

[33] Shang X, Huang C, Chang Q, Zhang L, Huang T (2010). Association between the ACTN3 R577X polymorphism and female endurance athletes in China. Int J Sports Med.

[34] Tiret L, Rigat B, Visvikis S, Breda C, Corvol P, Cambien F, Soubrier F (1992). Evidence, from combined segregation and linkage analysis, that a variant of the angiotensin I-converting enzyme (ACE) gene controls plasma ACE levels. Am J Hum Genet.

[35] Taylor RR, Mamotte CD, Fallon K, van Bockxmeer FM (1999). Elite athletes and the gene for angiotensin-converting enzyme. J Appl Physiol.

[36] Tsianos G, Sanders J, Dhamrait S, Humphries S, Grant S, Montgomery H (2004). The ACE gene insertion/deletion polymorphism and elite endurance swimming. Eur J Appl Physiol.

[37] Vincent B, De Bock K, Ramaekers M, Van den Eede E, Van Leemputte M, Hespel P, Thomis MA (2007). ACTN3 (R577X) genotype is associated with fiber type distribution. Physiol Genomics.

[38] Wang G, Mikami E, Chiu LL, DE Perini A, Deason M, Fuku N, Miyachi M, Kaneoka K, Murakami H, Tanaka M, Hsieh LL, Hsieh SS, Caporossi D, Pigozzi F, Hilley A, Lee R, Galloway SD, Gulbin J, Rogozkin VA, Ahmetov II, Yang N, North KN, Ploutarhos S, Montgomery HE, Bailey ME, Pitsiladis YP (2013). Association analysis of ACE and ACTN3 in elite Caucasian and east Asian swimmers. Med Sci Sports Exerc.

[39] Woods D, Hickman M, Jamshidi Y, Brull D, Vassiliou V, Jones A, Humphries S, Montgomery H (2001). Elite swimmers and the D allele of the ACE I/D polymorphism. Hum Genet.

[40] Yang N, Garton F, North K (2009). Alpha-actinin-3 and performance. Med Sports Sci.

[41] Yang N, MacArthur DG, Gulbin JP, Hahn AG, Beggs AH, Easteal S (2003). North K.ACTN3 genotype is associated with human elite athletic performance. Am J Hum Genet.

[42] Zhao B, Moochhala SM, ThamSY, Lu J, Chia M, Byrne C, Hu Q, Lee LK (2003). Relationship between angiotensin-converting enzyme ID polymorphism and VO2max of Chinese males. Life Sci.

